# Efficient design of partially nested randomized trials: A maximin approach

**DOI:** 10.1177/09622802251409388

**Published:** 2026-03-13

**Authors:** Math JJM Candel, Gerard JP van Breukelen

**Affiliations:** 1 Department of Methodology and Statistics, Care and Public Health Research Institute (CAPHRI), Maastricht University, Maastricht, The Netherlands; 2 Department of Methodology and Statistics, Graduate School of Psychology and Neuroscience, Maastricht University, Maastricht, The Netherlands

**Keywords:** Efficient design, individually randomized group treatment trials, partially nested randomized trials, power, sample size calculation

## Abstract

For two-treatment randomized trials with clustering in one of the treatment arms and a continuous outcome, designs are presented that minimize the number of subjects or the amount of research budget, when aiming for a desired power level. These designs optimize the treatment-to-control allocation ratio of study participants but also optimize the choice between the number of clusters (such as therapy groups) versus the number of persons per cluster (therapy group) in the arm with clustering. Optimal designs require prior knowledge of parameters from the analysis model, which are unknown during the design stage. We present maximin designs which address this by ensuring a pre-specified power level for plausible ranges of the unknown parameters, while maximizing the power for worst-case values of these parameters. Maximin designs are also derived when the number of clusters, or the cluster size is fixed due to practical constraints. An empirical example illustrates how to calculate sample sizes for such practical designs and shows how much these maximin designs can reduce the required research budgets compared to designs with equal subject numbers in treatment and control. A user-friendly R Shiny app facilitates these sample size calculations.

## 1 Introduction

**Figure 1.**
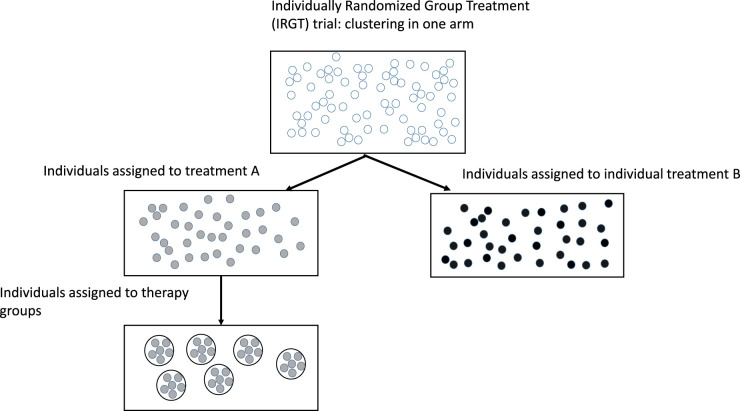
Graphical display of an Individually Randomized Group Treatment trial, with individuals assigned to six therapy groups in one of the treatment arms.

In randomized trials observations can be correlated when prior to randomization individuals are nested within clusters, and these clusters are assigned treatments. Examples are persons nested within health centers, pupils nested within schools, or employees nested within companies. When whole clusters are assigned to treatments, these trials are known as cluster randomized trials.^1^ Clustering may also occur when individuals are assigned to treatments, but these treatments are given to groups of individuals.^2–4^ In such trials,^5,6^ interactions between persons within a group may lead to their outcomes being correlated. These trials are referred to as individually randomized group treatment (IRGT) trials.^6^ Clustering may occur exclusively in one of the treatment arms, for example, when comparing group therapy with a condition that lacks any form of intervention or with a condition involving only medication. Examples are a trial in which patients with chronic musculoskeletal pain either receive usual treatment supplemented by participating in a learning program given in group sessions versus individual treatment with medication only,^7^ or a trial where tinnitus patients either receive group-based cognitive behavioral therapy addressing their dysfunctional cognitions versus receiving no treatment at all.^8^ Figure 1 displays an IRGT trial with groups of size 6 in treatment arm *G*, and with no clustering in treatment arm *I*.

Clustering effects can also arise from a treatment that is administered individually rather than to a group. If multiple patients receive treatment from the same therapist, these patients are likely to receive a more similar treatment compared to patients treated by different therapists. This therapist-related impact can result in correlated observations from different patients having the same therapist.^6,9^ Also in such scenarios, clustering may occur exclusively in one of the two treatment arms, for instance, when comparing psychotherapy with medication,^10^ or with a waiting-list condition.^11–13^ This design is also represented by Figure 1, where the therapy groups are actually persons sharing the same therapist, and the group size represents the caseload of a therapist.

For trials an important aim is to choose a design such that a sufficient power level is achieved for a test on the treatment effect. For designs with partial nesting, this involves choosing an appropriate number of groups and group sizes for one treatment, and an appropriate number of persons for the other treatment. When testing the null hypothesis of no treatment effect against the alternative hypothesis of a treatment effect, we perform a two-tailed test, and the variance of the estimated treatment effect estimate, $\;var(\hat{effect})$ , is (approximately) related to the power of the statistical test of the treatment effect as follows:

(1) $$\begin{array}{r} \frac{{\left(treatment\;effect\right)}^{2}}{var(\hat{effect})}\approx (z_{1-\alpha /2}+z_{1-\beta }{)}^{2}. \end{array}$$

Here $z_{1-\alpha /2}$ is the 100(1−α/2) th percentile of the standard normal distribution, defined by α , the significance level of the statistical test, and $z_{1-\beta }$ is the 100(1−β) th percentile of the standard normal distribution, determined by (1−β) , the power of the test. As equation (1) shows, everything else kept constant, the smaller the variance of the treatment effect estimate, the larger $z_{1-\beta }$ and thus (1−β) , the power of the test.

The variance of the treatment effect estimate is also related to the width of a confidence interval for the treatment effect. For a 100(1−α/2)% confidence interval, this width is (approximately) given by $2\;\times z_{1-\alpha /2}\times \sqrt{var(\hat{effect})}$ . The confidence interval expresses the precision of the treatment effect estimate: the smaller the interval, the more precise the effect estimate is.

Study design optimization entails different types of optimality, each determined by a different optimization criterion. For an overview, see Berger et al.^14^ and Atkinson et al.^15^ This paper targets maximizing the power of the test of the treatment effect by deriving designs that minimize the variance of the treatment effect estimate for a given research budget. Designs minimizing the variance of an estimate of a single parameter, like the treatment effect, are termed *c*-optimal designs. As shown in equation (1), minimizing the estimate's variance maximizes the power of the statistical test. Since the variance of the treatment effect estimate is proportional to the width of the confidence interval, these *c*-optimal designs also minimize, for a given research budget, the width of the confidence interval for the treatment effect. It should be noted that the optimal designs also minimize the research budget needed for a pre-specified power of a test on the treatment effect, or for a required precision of the effect estimate. If another design existed that attained the same power or precision of the effect estimate with a smaller research budget, then, since the variance of the treatment effect decreases with increasing budget, the design derived for the given research budget would not be optimal.^16^

The more a design minimizes the variance of the treatment effect estimate for a given research budget, or minimizes the budget needed for a given variance and thus given power and precision, the more efficient the design is. Now, it is well known that a cluster randomized trial is less efficient than an individually randomized trial, especially as the dependency of the observations within a cluster increases and the number of individuals per cluster grows.^17–19^ A trial with clustering in one arm lies between these two designs in terms of efficiency. A trial with clustering in only one arm typically arises when one treatment is administered in groups and the other individually, or when one treatment is delivered by therapists while the other involves only medication or no treatment at all. In such cases, researchers generally are not able to choose the most efficient one of these three designs.

Finding an optimal design for the trials considered in this paper involves determining how many subjects to allocate to each treatment, and for the arm with clustering, how many clusters or therapy groups versus how many subjects within each cluster or group to include. A complication is that both the value of $var(\hat{effect})$ and the optimal design depend on several variance parameters of the model that will be used in analyzing the data but are unknown in the design stage of a trial. As the optimal design is only optimal for specific values of those unknown parameters, not for the entire range of possible values, this is known as the local optimality problem.

Several approaches to address local optimality have been proposed in the literature. The Bayesian approach starts from a prior distribution for the unknown parameters and by repeatedly drawing from the prior, one can calculate the mean, median, or other desired percentile of the power.^20^ In this approach the design is chosen which maximizes the average power or some power percentile. This process is computationally time-intensive and yields a design that does not guarantee the required power level for an individual trial. In adaptive designs one starts with a predefined design, followed by intermediate analyses to update guesses about relevant model parameters and adapt the design accordingly.^21^ For cluster randomized trials, proposals for adapting the number of clusters^22^ or the sample size within each cluster^23^ have been examined. More recent studies considered design adaptions that take care of the uncertainty in the estimates obtained in the interim analysis, in a frequentist approach^24^ or in a Bayesian approach updating a prior in the interim analysis.^25^ These approaches require a duration of the trial that is sufficient for conducting intermediate analyses and subsequently modifying the design.

In this paper, we will take a rather simple approach, known as the maximin approach.^17^ Deriving a maximin design involves four steps:

1. Specify plausible ranges for those parameters of the analysis model on which $var(\hat{effect})$ depends.
2. Given a research budget, specify the set of feasible designs.
3. For each design find the parameter values within their plausible ranges which maximize $var(\hat{effect})$ , and thus minimize the efficiency of that design.
4. Choose the design that minimizes the maximum (worst-case) $var(\hat{effect})$ , as obtained in step 3, and thus the design that maximizes the minimum efficiency.

The resulting design is called the maximin design, which, for a given research budget, is the optimal design for the worst-case scenario, as defined by the set of parameter values chosen in step 3. The maximin design offers the advantage of not only maximizing efficiency, power and estimation precision in the worst-case scenario but also ensuring at least the same efficiency, power and precision for all other plausible parameter values. So, for all other parameter values than the worst-case values chosen in step 3, the variance of the effect estimate is smaller, and the power for hypothesis testing and the precision of estimating this effect is larger.

Instead of considering the efficiency of a design, the maximin approach can also be employed with a relative efficiency criterion: the variance of the estimated treatment effect under the optimal design relative to the variance of the estimated treatment effect under the design that is being considered. For each feasible design, this relative efficiency is then first determined as a function of each parameter vector in the parameter space because the optimal design itself varies across the parameter space. Comparable to step 3 above, for each feasible design, the smallest relative efficiency is then obtained across the parameter space. Then, comparable to step 4 above, of all feasible designs, that design is selected that maximizes this minimum relative efficiency.^14,26^ This design is safe in that it stays as close to the optimal design as possible over the whole range of plausible parameter values. If the design's minimum relative efficiency is close to 1, then it can be considered a robust design. Such a design may be different from the design that maximizes the minimum efficiency.^27^ The efficiency approach may yield a design that may be much less efficient than the optimal design for some of the parameter values in their plausible ranges. Also, the maximin design based on efficiency, as in this paper, may turn out to be optimal at the boundary values of parameter ranges – values that may not be most plausible. This overemphasis on an unlikely scenario may lead to a large research budget. But, on the other hand, employing a relative efficiency criterion is not safe in that it does not yield a design that guarantees a desired power level across the whole parameter space. Since a maximin approach based on efficiency is safe in that sense, we adopt this approach in this paper.

In this paper, we will examine two-treatment parallel trials with nesting in one of the arms. We will derive optimal and maximin designs and will also consider when practical constraints fix the total number of groups or therapists or the size of groups or the caseload per therapist. We propose a linear mixed model for analysis, allowing for different outcome variances as well as different costs across treatments, thus presenting rather general optimal and maximin designs.

We will show how to calculate sample sizes for maximin designs with a real example. While the optimal and maximin designs assume group sizes or therapist caseloads within a treatment to be equal, real-world scenarios often involve varying group sizes and caseloads. Even if one recruits an equal number of individuals for each included group or for each therapist, dropout may lead to varying group sizes and caseloads in the data analysis phase. This results in efficiency and power loss.^28^ We will address how to restore efficiency due to varying group sizes and caseloads. Below, we first present the model for the analysis of a parallel trial with clustering in one arm and then move on to the optimal and maximin designs.

## 2 Linear mixed model for data analysis

Let treatment *G* be the condition with clustering (*G* for groups) and treatment *I* be the condition without clustering (*I* for individuals). If there are *K* clusters in treatment arm *G* and there are $n_{G}$ members in each cluster, then in treatment *G*, a person is indexed by *i* = 1,…, $n_{G}$ and a cluster by *j* = 1,…*K.* In the arm without clustering a person is indexed by *i* = 1,…, $n_{I}$ *,* there being $n_{I}$ persons in this arm, and to distinguish a person in arm *I* from a person in arm *G*, *j* is set to *K* + 1 in arm *I,* not being the index for a cluster in that arm. The following mixed regression model is a suitable model for outcome $y_{ij}$ :

(2) $$\begin{array}{r} y_{ij}=\beta_{0}+U_{0j}x_{ij}+\;\beta_{1}x_{ij}+\varepsilon_{ijG}x_{ij}+\varepsilon_{ijI}(1-x_{ij})\;. \end{array}$$

Here $x_{ij}=1\;$ if a person belongs to the arm with clusters, treatment *G*, and $x_{ij}=0$ otherwise. The parameter $\beta_{0\;}$ is the mean outcome in treatment *I* and $\;\beta_{1}$ is the treatment effect. By adding a random effect *U_0j_* in treatment *G*, the outcome in this arm is allowed to vary between clusters (groups or therapists). The random effect *U_0j_* is normally distributed with mean zero and variance $\sigma_{0}^{2}$ . Further, $\varepsilon_{ijG}$ and $\varepsilon_{ijI}\;$ reflect subject and measurement error effects in treatment *G* and *I*, are independent of *U_0j_*, and are normally distributed with mean zero and variances $\sigma_{\varepsilon G}^{2}$ and $\sigma_{\varepsilon I}^{2}\;$ for treatment *G* and *I* respectively. Due to the common cluster effect $U_{0j}$ , outcomes of two persons in the same cluster in treatment *G* are correlated, and this intracluster correlation is $\rho =\sigma_{0}^{2}/(\sigma_{0}^{2}+\sigma_{\varepsilon G}^{2})=\sigma_{0}^{2}/\sigma_{G}^{2}$ , where $\sigma_{G}^{2}$ is the total outcome variance in treatment *G*. Due to the absence of a random effect $U_{0j}$ in treatment *I*, outcomes of persons in that arm are not correlated. The ratio of outcome variances in the cluster condition versus the individual condition is denoted by $\psi =(\sigma_{0}^{2}+\sigma_{\varepsilon G}^{2})/\sigma_{\varepsilon I}^{2}.\;$ The model in equation (2) extends the model adopted by Heo et al.^29^ in deriving sample size calculation formulas, by allowing for heterogeneity in the individual-level variance.

This design and associated analysis model are special cases of the design and model for cluster randomized trials with clusters in both arms, in that the intraclass correlation ρ for one of the arms is 0. With this restriction, the variance of the estimated treatment effect, ${\hat{\beta }}_{1},$ follows from that of a cluster randomized trial, given by, for instance Van Breukelen et al.,^30^ but was also explicitly derived by Moerbeek et al.^31^:

(3) $$\begin{array}{r} var({\hat{\beta }}_{1})=\left[((n_{G}-1)\rho +1)\;\frac{\psi }{n_{G}K}+\frac{1}{n_{I}}\right]\sigma_{\varepsilon I}^{2}. \end{array}$$

## 3 Optimal and maximin design

Since optimal designs minimize the variance of the treatment effect estimate for a given research budget, a budget function has to be defined. Let *c* be the costs for including a cluster (e.g. group or therapist) in treatment arm *G*, and, similarly, let $s_{G}$ and $s_{I}$ be the costs attached to treating and measuring subjects within treatment *G* and *I* respectively. A plausible budget function is:

(4) $$\begin{array}{r} b=K(c+n_{G}s_{G})+n_{I}s_{I}. \end{array}$$

Moerbeek et al.^31^ derived the optimal ratio of the total number of subjects in treatment *G,* $Kn_{G}$ *,* versus the number of subjects in treatment *I*, $n_{I}$ . However, the full optimal design with specification of *K* and $n_{G}$ instead of their product can be shown to be as follows (see Appendix A):

(5) $$\begin{array}{r} \begin{array}{rl} n_{G}^{opt} & =\sqrt{\frac{c}{s_{G}}\times \frac{\left(1-\rho \right)}{\rho }},\;K^{opt}=b\times \frac{\sqrt{\rho \psi }}{\sqrt{c}\left(\left(\sqrt{\rho }\sqrt{c}+\sqrt{\left(1-\rho \right)}\sqrt{s_{G}}\right)\sqrt{\psi }+\sqrt{s_{I}}\right)},\;\text{and}\\ n_{I}^{opt} & =b\times \frac{1}{\sqrt{s_{I}}\left(\left(\sqrt{\rho }\sqrt{c}+\sqrt{\left(1-\rho \right)}\sqrt{s_{G}}\right)\sqrt{\psi }+\sqrt{s_{I}}\right)}. \end{array} \end{array}$$

Substituting these optimal sample sizes into equation (3) gives the variance of the treatment effect estimate for the optimal design. From equation (5) it follows that the optimal allocation ratio of persons to the two treatments is given by:

(6) $$\begin{array}{r} \frac{n_{G}^{opt}K^{opt}}{\text{\textbackslash{}; }n_{I}^{opt}}=\sqrt{\frac{c}{s_{G}}\times \frac{\left(1-\rho \right)}{\rho }}\;\times \frac{\sqrt{\rho \psi }}{\sqrt{c}}\times \sqrt{s_{I}}=\sqrt{\frac{s_{I}}{s_{G}}\times (1-\rho )\times \psi }\;=\sqrt{\frac{s_{I}}{s_{G}}}\;\times \frac{\sigma_{\varepsilon G}}{\sigma_{\varepsilon I}}, \end{array}$$

which is like the optimal allocation ratio of persons in a standard two-treatment randomized trial without nesting, with no cluster costs being involved and no random intercept variance of treatment *G*.^17^ Section 1 of the Supplemental materials shows that the allocation ratio in equation (6) is actually a function of the variance of the cluster means in *G*, the variance of individual scores in *I*, and the cluster- and subject specific costs, which reduces to the expression in equation (6).

The optimal design in equation (5) and the variance of $\hat{\beta_{1}}\;$ in equation (3) depend on the parameters $\sigma_{\varepsilon I}^{2}$ , ψ , and ρ , on which there will be only limited knowledge. From equation (3) the variance of $\hat{\beta_{1}}\;$ increases as a function of $\sigma_{\varepsilon I}^{2}$ , ψ , and ρ . So the maximin design and the variance of the effect estimate of the maximin design are obtained by choosing the largest values for these parameters within their plausible ranges, $\sigma_{\varepsilon I\;max}^{2}$ , $\psi_{max}$ , and $\rho_{max}$ , and substituting these into equations (5) and (3) respectively. To obtain the required budget in equation (5), the variance of the effect estimate for the maximin design is combined with equation (1). If the effect size is defined as $\delta =\beta_{1}/\sigma_{\varepsilon I}$ , the treatment effect relative to the standard deviation of the outcome for treatment *I,* then, instead of choosing the largest value for $\sigma_{\varepsilon I}^{2}$ within its plausible range, one can also choose the smallest relevant effect size, $\delta_{min}$ . Combining equations (1), (3), and (5), the budget required to have 1−β power to detect an effect of size $\delta_{min}$ in a two-tailed test at type I error rate α can now be calculated as:

(7) $$\begin{array}{r} b=\frac{{\left(z_{1-\alpha /2}+z_{1-\beta }\right)}^{2}}{\delta_{min}^{2}}\times {\left(\left(\sqrt{c}\sqrt{\rho_{max}}+\sqrt{s_{G}}\sqrt{(1-\rho_{max})\;}\right)\sqrt{\psi_{max}}+\sqrt{s_{I}}\right)}^{2}. \end{array}$$

Substituting the calculated budget *b* into equation (5), then yields the number of clusters, $K^{mmd}$ , and the sample size for treatment *I,* $n_{I}^{mmd}$ , of the maximin design.

## 4 Fixed cluster sizes

In some cases, the cluster size for treatment *G* will be (more or less) fixed, for instance, in case of group therapy, where there may be an ideal group size or, in case of multiple persons being assigned to the same therapist, there may be an ideal caseload. In these cases, $n_{G}$ is fixed, and the optimal number of clusters for treatment *G* and the optimal number of persons for treatment *I* can be derived as (see Appendix A):

(8) $$\begin{array}{r} \begin{array}{rl} K^{opt} & =b\times \frac{\sqrt{\psi }\sqrt{\rho (n_{G}-1)+1}}{\left(\sqrt{c+n_{G}s_{G}}\right)\left(\left(\sqrt{c+n_{G}s_{G}}\right)\left(\sqrt{\psi }\sqrt{\rho (n_{G}-1)+1}\right)+\sqrt{n_{G}s_{I}}\right)},\;\text{and}\\ n_{I}^{opt} & =b\times \frac{\sqrt{n_{G}}}{\sqrt{s_{I}}\left(\left(\sqrt{c+n_{G}s_{G}}\right)\left(\sqrt{\psi }\sqrt{\rho (n_{G}-1)+1}\right)+\sqrt{n_{G}s_{I}}\right)}. \end{array} \end{array}$$

By substituting equation (8) into equation (3), we obtain the variance of the effect estimate for the optimal design.

**Figure 2.**
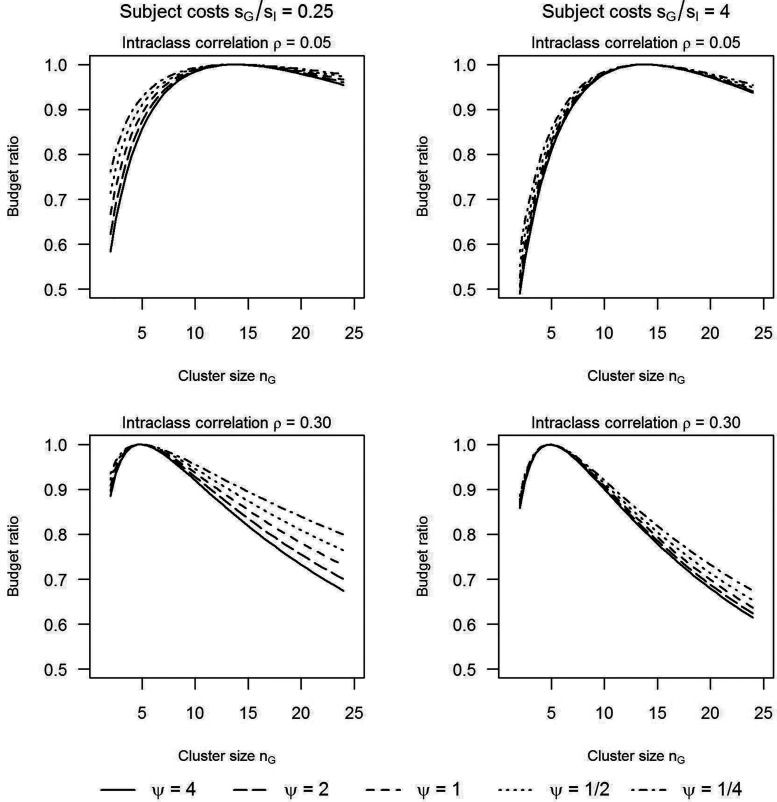
Ratio of the budget of an unrestricted maximin design versus the budget of a maximin design with the cluster size in treatment G fixed at a value on the horizontal axis.

The maximin design and its variance of the effect estimate is again obtained by choosing the largest values for $\sigma_{\varepsilon I}^{2}$ , ψ and ρ within their plausible ranges and substituting these into equations (8) and (3) respectively. When combined with equation (1), the budget needed for determining the maximin design can be calculated as, with $\beta_{1}/\sigma_{\varepsilon I\;max}\;$ translated into $\delta_{min}$ :

(9) $$\begin{array}{r} b=\frac{{\left(z_{1-\alpha /2}+z_{1-\beta }\right)}^{2}}{\delta_{min}^{2}}\times \frac{1}{n_{G}}{\left(\left(\sqrt{\psi_{max}}\sqrt{\rho_{max}(n_{G}-1)+1}\right)\left(\sqrt{c+n_{G}s_{G}}\right)+\sqrt{n_{G}s_{I}}\right)}^{2}. \end{array}$$

It is instructive to explore how much more budget is required if $n_{G}$ is fixed, relative to a design in which $n_{G}$ is optimally chosen. Figure 2 shows the budget ratio, that is, the budget of a maximin design without restrictions on $n_{G}$ relative to the budget of the maximin design in which $n_{G}$ is fixed. Four scenarios are examined in Figure 2. The left column is for a cost ratio $s_{G}/s_{I}=0.25$ and the right column for a cost ratio $s_{G}/s_{I}=4$ . The upper row is for an intraclass correlation ρ=0.05 and the lower row for an intraclass correlation ρ=0.30 . In all scenarios $c/s_{G}=10.$ Within each row the maximin choice for $n_{G}$ is the same and corresponds with the value on the horizontal axis where the budget ratio is exactly 1. As can be seen, since the budget ratio of a free relative to a fixed $n_{G}$ can be much smaller than 1, the budget that is required when $n_{G}$ is fixed may be substantially larger than the budget of the maximin design where $n_{G}$ is chosen optimally. Furthermore, if the variance ratio ψ increases, the budget required by the design with a suboptimal $n_{G}$ relative to the budget required by the maximin design increases (Section 2 of the Supplemental materials contains a proof). This can be understood by noting that, if ψ increases, a larger part of the total budget needs to be allocated to treatment *G* (see equations (5) and (8)).

### 4.1 Maximin versus balanced design with restrictions on cluster sizes: Empirical illustration

In practice, as in group therapy, cluster sizes cannot always be chosen freely. Figure 2 shows that suboptimal choices for $n_{G}$ may significantly increase the research budget. However, group therapy often allows a range of feasible cluster sizes. Many trials have approximately equal numbers in both treatments,^4,8,32^ so it is instructive to compare the budget for a balanced design to that for a maximin design when $n_{G}$ is restricted. Consider replicating Conrad et al.,^8^ which studied cognitive behavioral treatment effects on dysfunctional cognitions in chronic tinnitus patients. The original study compared three treatments, but here we focus on group-based cognitive behavioral therapy versus an active control involving participation in an internet-based forum. Conrad et al.^8^ considered three main outcomes: tinnitus-related dysfunctional cognitions, catastrophic thinking, and avoidance cognitions. The variance ratios ψ post-intervention for the group-based treatment versus control across the three outcomes ranged from 0.71 to 1.18, so we assume $\psi_{max}=1.5$ for the maximin design. Conrad et al.^8^ did not provide intraclass correlations, but in group interventions, these typically range from 0.01 to 0.30.^2,4,33,34^ For the maximin design, we set $\rho_{max}=0.3$ .

Suppose that for the cognitive behavioral treatment feasible values for the size of the groups, $n_{G}$ , are between 4 and 10, and that we want to detect a medium sized effect, that is, $\delta =\beta_{1}/\sigma_{\varepsilon I}=0.5,$ in a two-tailed test with 80% power at a 5% type I error rate. This actually is a scenario in between a maximin design in which one is free to choose $n_{G}$ and a maximin design where $n_{G}$ is fixed at one specific value. No costs are specified, but commonly the costs at the group level, *c*, will be higher than the costs of including a subject within a group, $s_{G}.$ We consider $c/s_{G}\in \{5,10\}.\;$ In the study by Conrad et al.^8^ there will be more costs involved per subject when participating in group therapy compared to being in the control condition, where no therapy at all is given, so $c/n_{G}+s_{G}>\;s_{I}$ , or $c/(s_{G}n_{G})+1>s_{I}/s_{G}$ . So, suppose that even if $n_{G}=10$ , the cost per subject (including cluster costs) for treatment *G* is larger than that for *I.* Then, if $\;c/s_{G}=5$ , we have $\;s_{G}/s_{I}>2/3.$ Similarly, if $c/s_{G}=10$ , then $\;s_{G}/s_{I}>1/2$ . In other trials the costs per individual in treatment *I* may be larger than the cost per individual in treatment *G*, such as when a rather expensive drug is used for treatment *I*. So, we also allow for cost scenarios such that $c/(s_{G}n_{G})+1\;<s_{I}/s_{G}$ (see second column of Table 1).

**Table 1.** Number of groups *K*, group size $n_{G}$ , with $n_{G}\;\in \;\{4,5,6,7,8,9,10\},\;$ and number of subjects in the individual condition $n_{I}$ needed by the maximin design (MMD) and balanced design (BD) (in which $Kn_{G}=n_{I}$ ) of the group-based cognitive behavioral treatment trial, for a power of 80% to detect a treatment effect of size δ = 0.50, with α=5 % two-tailed, for various cost ratios $c/s_{G}$ and $\;s_{G}/s_{I}$ and for $\psi_{max}=1.5$ and $\rho_{max}=0.3$ .

$c/s_{G}$	$s_{G}/s_{I}$	*Maximin Design*^a^ (*K*, $n_{G},n_{I})$	*Balanced Design*^b^ (*K*, $n_{G},n_{I})$	*Budget reduction of MMD relative to BD*
5	0.1	(51, 4, 57)	(31, 4, 124)	32.3%
5	0.25	(41, 4, 72)	(31, 4, 124)	15.2%
5	0.5	(35, 4, 88)	(31, 4, 124)	6.8%
5	2	(29, 4, 144)	(31, 4, 124)	2.3%
5	4	(27, 4, 191)	(31, 4, 124)	6.2%
5	10	(26, 4, 283)	(25, 6, 150)	9.6%
10	0.1	(42, 5, 63)	(31, 4, 124)	24.7%
10	0.25	(34, 5, 81)	(31, 4, 124)	10.3%
10	0.5	(33, 4, 102)	(31, 4, 124)	2.3%
10	2	(26, 5, 172)	(25, 6, 150)	−0.2%
10	4	(26, 4, 230)	(25, 6, 150)	3.7%
10	10	(23, 5, 344)	(25, 6, 150)	8.6%

^a^ $n_{I}\;$ and *K* were rounded up to the nearest integer.

^b^ *K* was calculated by combining equations (1) and (3) such as to yield 80% power and was rounded up to the nearest integer, giving $n_{I}=Kn_{G}$ for a fixed $n_{G}$ .

The maximin design can be determined as follows: For each $n_{G}\;\in \;\{4,5,6,7,8,9,10\},$ the budget *b* is determined according to equation (9). Substituted into equation (8) this then yields *K* and $n_{I}$ . For each $n_{G}$ , $n_{I}$ and *K* are rounded up to the nearest integer. Of the resulting seven designs the design $\left(K,n_{G},n_{I}\right)$ that requires the smallest budget is displayed as the maximin design in Table 1. For different cost scenarios the maximin design is compared with a balanced design in which the number of persons in both treatment conditions is the same. For the balanced design, for each $n_{G}\in \{4,5,6,7,8,9,10\},\;K$ and thus also $\;n_{I}(=Kn_{G})$ are calculated according to equation (1) and equation (3) such as to yield 80% power. Rounding *K* up to the nearest integer, then also yields the number of persons in the individual condition, since $n_{I}=Kn_{G}.\;$ Of the resulting seven balanced designs $\left(K,n_{G},n_{I}\right)$ the design that requires the smallest budget is shown in Table 1.

For the maximin design it follows from equation (8) and equation (9) that for the same $n_{G}$ , as $\;s_{G}/s_{I}$ increases while $c/s_{G}$ does not change, more persons are assigned to the individual treatment condition (treatment *I*) and fewer groups, and thus fewer persons, to the group condition (treatment *G*). For the example in Table 1, this implies that for $c/s_{G}=5,\;$ with increasing $\;s_{G}/s_{I}\;$ the maximin design first becomes more balanced, leading to a smaller budget reduction of the maximin design as compared to the balanced design, until $\;s_{G}/s_{I}=2$ , after which the design again becomes more unbalanced in the other direction, leading again to a larger budget reduction of the maximin design. For $c/s_{G}=10$ , although $n_{G}$ of the maximin design then varies a bit, when $\;s_{G}/s_{I}$ increases, a similar pattern occurs.

## 5 Fixed number of clusters

In case therapists treat multiple patients individually, there may be a fixed or limited number of therapists, or, in case a therapist carries out group therapy there may be maximum number of groups that practically can be handled. In such a case *K* is fixed, and one can determine the optimal values for $n_{G}$ and $n_{I}$ (see Appendix A for a proof) as:

(10) $$\begin{array}{r} n_{G}^{opt}=\frac{(b-Kc)\sqrt{1-\rho }\sqrt{\psi }}{K\sqrt{s_{G}}\left(\sqrt{s_{G}}\sqrt{1-\rho }\sqrt{\psi }+\sqrt{s_{I}}\right)},\text{\textbackslash{}; \textbackslash{}; and\textbackslash{}; \textbackslash{}; }n_{I}^{opt}=\frac{\left(b-Kc\right)}{\sqrt{s_{I}}\left(\sqrt{s_{G}}\sqrt{1-\rho }\sqrt{\psi }+\sqrt{s_{I}}\right)}\text{\textbackslash{}; }. \end{array}$$

Substituting $n_{G}^{opt}$ and $n_{I}^{opt}$ into equation (3) yields the variance of the treatment effect estimator for the optimal design in case of a fixed number of clusters.

The maximin design and associated variance of the treatment effect estimate are again obtained by choosing the largest values for $\sigma_{\varepsilon I}^{2}$ , ψ and ρ within their plausible ranges and substituting these into equations (10) and (3) respectively. When combined with equation (1), the budget needed for having sufficient power of the maximin design can be calculated as:

(11) $$\begin{array}{r} b=\frac{{\left(z_{1-\alpha /2}+z_{1-\beta }\right)}^{2}K{\left(\sqrt{\psi_{max}}\sqrt{1-\rho_{max}}\sqrt{s_{G}}+\sqrt{s_{I}}\right)}^{2}}{\delta_{min}^{2}K-\rho_{max}\psi_{max}{\left(z_{1-\alpha /2}+z_{1-\beta }\right)}^{2}}+Kc. \end{array}$$

If the cluster sizes of the maximin design required for a certain power level are too large, for instance, as this group size in group therapy is not feasible, one either has to accept a lower power level or a larger effect size for the study. One could also try to slightly increase the number of groups, *K*. Finally, note that when the number of clusters is fixed, a desired power level may not always be obtained, since the variance of the treatment effect estimate has a lower bound. Specifically, even if $n_{G}$ and $n_{I}$ go to infinity, the variance of the effect estimate in equation (3) cannot be lower than $\rho \sigma_{G}^{2}/K,$ which, for a non-zero intraclass correlation for treatment *G*, limits the power that can be realized with a fixed and finite *K*.

**Figure 3.**
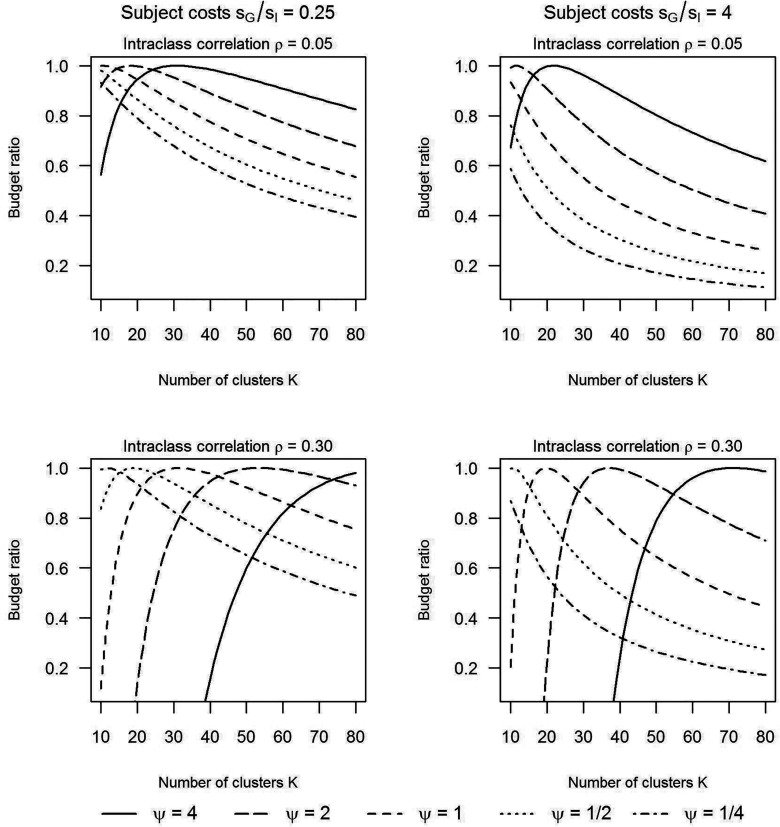
Ratio of the budget of an unrestricted maximin design versus the budget of a maximin design with the number of clusters in treatment G fixed at a value on the horizontal axis.

Let's also consider how much more budget is required if the number of clusters *K* is fixed compared to a design in which *K* is chosen optimally. For the same scenarios as in Figure 2, Figure 3 shows the budget ratio, that is, the budget required by a maximin design without restrictions on *K* relative to the budget required by a maximin design in which *K* is fixed. In all scenarios $c/s_{G}=5$ . Note that the optimal number of clusters is dependent on all parameters that are varied: the value of *K* where the budget ratio equals 1, is, in each of the four plots and for each of the four curves in each plot at another location on the horizontal axis. Since the budget ratios can become much smaller than 1, the additional budget that may be required for a maximin design with *K* fixed compared to a maximin design where *K* is chosen optimally, may be substantial.

### 5.1 Maximin versus balanced design with restrictions on the number of clusters: Empirical illustration

Returning to the example in Table 1, suppose that the clusters do not represent therapy groups, but caseloads of different therapists who give cognitive behavioral therapy on an individual basis. Because of a limited pool of therapists, there is a maximum of 22 therapists in the trial, so that a maximin design must be determined with restrictions on *K.* The same cost scenarios are considered as in Table 1. Also assume that $\psi_{max}=1.5$ and $\rho_{max}=0.3$ , and, similar to the example in Table 1, we want to detect an effect size $\delta_{min}=\beta_{1}/\sigma_{\varepsilon I}=0.5$ , with 80% power in a two-tailed test at a 5% type I error rate. When $n_{G}$ and $n_{I}$ approach infinity, the ratio of the squared treatment effect and the variance of the treatment effect estimate approach $K\delta_{min}^{2}/(\psi_{max}\rho_{max})$ , so that, by equation (1), at least *K* *=* 15 therapists are required.

Column 3 of Table 2 contains the number therapists of the maximin design if there are no restrictions on *K.* Column 4 displays the maximin design where *K* is at most 22, whereas column 5 displays the balanced design with the same restrictions on *K*. Since the maximum feasible number of therapists is, for each cost scenario, lower than the maximin number of therapists if there are no restrictions on *K* (see column 3 of Table 2), *K* = 22 is always the choice that minimizes the required research budget (see column 4 of Table 2). Furthermore, for a fixed *K*, as $\;s_{G}/s_{I}$ increases, the cluster size $n_{G}$ decreases, whereas $n_{I}$ increases. Note that for restrictions on the cluster size $n_{G}$ (Table 1) as well as for restrictions on the number of clusters *K* (Table 2), the effects of the cost ratio $\;s_{G}/s_{I}$ on the allocation ratio are the same as for the classical randomized controlled trial^17^ and the classical cluster randomized trial^30^: more persons are allocated to the cheaper treatment because, for the price of giving one person the more expensive treatment, more than one person can be given the cheaper treatment, thus increasing the total sample size.

**Table 2.** Number of therapists *K*, with K∈{15,16,17,18,19,20,21,22} , group size $n_{G}$ and number of subjects in the individual condition $n_{I}$ needed by the maximin design (MMD) and balanced design (BD) (in which $Kn_{G}=n_{I}$ ) of the group-based cognitive behavioral treatment trial, for a power of 80% to detect a treatment effect of size δ = 0.50, with α= 5% two-tailed, for various cost ratios $c/\;s_{G}$ and $\;s_{G}/s_{I}$ and for $\psi_{max}=1.5$ and $\rho_{max}=0.3$ .

$c/s_{G}$	$s_{G}/s_{I}$	$K^{mmd}\;$ ^a^	*Maximin Design*^b^ (*K*, $n_{G},n_{I})$	*Balanced Design*^c^ (*K*, $n_{G},n_{I})$	*Budget reduction of MMD relative to BD*
5	0.1	54	(22, 18, 117)	(22, 9, 198)	26.7%
5	0.25	43	(22, 13, 116)	(22, 9, 198)	14.2%
5	0.5	38	(22, 10, 152)	(22, 9, 198)	9.9%
5	2	31	(22, 8, 215)	(22, 9, 198)	3.3%
5	4	29	(22, 7, 268)	(22, 9, 198)	7.4%
5	10	27	(22, 6, 373)	(22, 9, 198)	14.8%
10	0.1	43	(22, 18, 117)	(22, 9, 198)	25.5%
10	0.25	35	(22, 13, 116)	(22, 9, 198)	14.2%
10	0.5	31	(22, 10, 152)	(22, 9, 198)	8.6%
10	2	26	(22, 8, 215)	(22, 9, 198)	2.6%
10	4	25	(22, 7, 268)	(22, 9, 198)	5.7%
10	10	24	(22, 6, 373)	(22, 9, 198)	11.1%

^a^ Number of clusters in the maximin design without restrictions on the number of clusters.

^b^ $n_{G}$ and $n_{I}$ were rounded up to the nearest integer.

^c^ $n_{G}$ was calculated by combining equations (1) and (3) such as to yield 80% power and was rounded up to the nearest integer, giving $n_{I}=Kn_{G}$ for a fixed *K.*

The maximin design is also compared with a balanced design in which the number of persons in both treatment conditions is the same. For the balanced design, for each *K* ∈{15,16,17,18,19,20,21,22} , $n_{G}$ and thus also $\;n_{I}(=Kn_{G})$ are calculated according to equation (1) and equation (3) such as to yield 80% power. Rounding $n_{G}$ up to the nearest integer, then also yields the number of persons in the individual condition, since $n_{I}=Kn_{G}$ . Of the resulting eight balanced designs $\left(K,n_{G},n_{I}\right)$ the design that requires the smallest budget is displayed in Table 2. As $\;s_{G}/s_{I}$ increases until 2, the maximin design approaches the balanced design, and the budget reduction of this design compared to the balanced design decreases. As $\;s_{G}/s_{I}$ increases further, the maximin design again becomes more unbalanced, and yields larger budget reductions compared to the balanced design. This is the same trend as for the case of restricted cluster size instead of restricted number of clusters (see Table 1).

## 6 Minimizing the sample size

Until now, we considered minimization of the study budget needed, either with, or without, constraints on the cluster size or the number of clusters. In some cases, one may want to minimize the total number of persons involved in a trial. This can be accommodated by setting $s_{G}=s_{I}=1$ and c=0 in the budget function in equation (4), yielding as budget $b=Kn_{G}+n_{I}=N$ , the total sample size. With these specifications of the subject-specific and cluster costs, the optimal cluster size in treatment arm *G* is 0 (see equation (5)), implying no data for this treatment arm, and so, the optimal cluster size becomes the smallest possible positive integer. This is because, for ρ > 0, increasing *K* reduces both the $\sigma_{0}^{2}$ part and the $\sigma_{\varepsilon G}^{2}$ part of the variance of the treatment effect estimate, whereas increasing $n_{G}\;$ only reduces the $\sigma_{\varepsilon G}^{2}$ part (see equation (A19) of Appendix A), which makes it optimal to choose $n_{G}$ as small as is practically feasible. For this smallest possible $n_{G}$ , the budget of the maximin design can then be determined by choosing the largest plausible values for ψ and ρ and the smallest relevant effect size $\delta_{min}$ in equation (9). By substituting the resulting budget into equation (8), the maximin values for *K* and $n_{I}$ are obtained. Now, suppose $\psi_{max}=1.5$ , $\rho_{max}=0.3$ , the smallest relevant effect $\delta_{min}=0.5$ as in Table 1, and the smallest feasible cluster size is $n_{G}=4$ . Then the maximin design can be calculated as $K^{mmd}=36$ and $n_{I}^{mmd}=85$ , whereas for a balanced design we have *K* = 31 and $n_{I}=124$ , implying a 7.7% smaller total sample size for the maximin design than for the balanced design (i.e. total *N* = 229 versus 248).

In case there are restrictions on the number of clusters *K*, where *K* can be 22 at most and we aim to minimize the total sample size, we also set $s_{G}=s_{I}=1$ and c=0 in equations (10) and (11). Choosing the largest plausible values for ψ and ρ , and the smallest relevant effect size $\delta_{min}$ in equation (11) yields the required budget, which then, upon substitution into equation (10), yields the maximin cluster size for arm *G* and the maximin number of persons in the arm without clustering. For $\psi_{max}=1.5$ , $\rho_{max}=0.3$ , and a smallest relevant effect $\delta_{min}=0.5$ , the maximin design is $K^{mmd}=22$ , $n_{G}^{mmd}=9$ , and $n_{I}^{mmd}=178$ , which reduces the sample size by 5.1% compared to that required for the balanced design (*K* = 22, $n_{G}=9$ , and $n_{I}=198$ ).

## 7 Interactive shiny app for sample size calculation

For individually^17^ and cluster randomized trials^35^ menu-driven interactive programs are available to calculate sample sizes for optimal and maximin designs. To also facilitate sample size calculation for maximin designs for trials with nesting in one arm, an R Shiny app^36^ has been developed: https://unimaasmc.shinyapps.io/Sample_size_PNRT_MMD/. In Table 1, calculations for each integer-valued $n_{G}$ in the feasible range rounded the maximin number of clusters *K* and the individual condition size $n_{I}$ up to the nearest integer. Similarly, in Table 2, for each integer-valued *K* in its feasible range, both $n_{G}$ and $n_{I}\;$ were rounded up to the nearest integer. This ensures each design meets the required power level. However, to optimize these designs further, in the Shiny app the power and costs for other nearby integer-valued configurations of *K*, $n_{G}$ and $n_{I}$ are checked. For the unconstrained design, the procedure involves systematically rounding two of the three design parameters, for instance *K* and $n_{G}$ , up or down using equation (7) and suitable expressions from equation (5) and recalculating the third parameter, $n_{I}$ , by equations (1) and (3), rounding it up to meet power requirements. This process is repeated by instead rounding *K* and $n_{I}$ up or down and recalculating $n_{G}$ , and again by rounding $n_{G}$ and $n_{I}$ up or down and recalculating *K*, each time using equation (7) and suitable expressions from equation (5) for the initial design parameters and using equations (1) and (3) for the third. This yields up to 12 candidate designs, from which the lowest-cost design is selected as the maximin design. A similar approach applies when numbers of clusters, *K*, or cluster sizes, $n_{G}$ , are already set to an integer value, as in Tables 1 and 2. For each *K* in its feasible range four designs with integer-valued cluster sizes $n_{G}$ and individual condition sizes $n_{I}$ need to be evaluated, and for each $n_{G}$ in its feasible range four designs with integer-valued numbers of clusters *K* and integer-valued sizes $n_{I}$ . The R-code of the Shiny app can be retrieved at the Open Science Framework from https://osf.io/p68gm/.

For the empirical illustration in Section 4 with $n_{G}\;\in \;\{4,5,6,7,8,9,10\}$ , $\psi_{max}=1.5$ , and $\rho_{max}=0.3$ , δ = 0.50, α=5% two-tailed, a power of 80%, consider $c/\;s_{G}=5$ and $\;s_{G}/s_{I}=10.$ Using the refined procedure in the Shiny app yields $\left(K,n_{G},n_{I}\right)$ = (25, 4, 299) as maximin design. This design reduces the required budget of the maximin design in which $n_{I}\;$ and *K* were rounded up to the nearest integer as in Table 1, that is, $\left(K,n_{G},n_{I}\right)$ = (26, 4, 283), by 2.8%. As another example, if *K* is a most 22, $\psi_{max}=1.5$ and $\rho_{max}=0.3$ , δ = 0.50, α=5% two-tailed, the power is 80%, and the goal is to minimize the total sample size, rounding $n_{G}$ and $n_{I}$ up to the nearest integer yielded $\left(K,n_{G},n_{I}\right)$ = (22, 9, 178) (see Section 6). The Shiny app provides (*K*, $n_{G},n_{I})$ = (22, 8, 185) as maximin design, reducing the total sample size from 376 to 361—a 4.0% decrease.

## 8 Correcting sample sizes for unknown variances and unequal cluster sizes

Sample sizes were determined using equation (1), which assumes a standard normal approximation for the test statistic. Adjustments are needed when estimating intraclass correlations and outcome variances. For non-varying cluster sizes, the treatment effect can also be assessed by an independent samples *t*-test comparing group or therapist means in one treatment to individual scores in the other. This implies that power according to the *t*-distribution can be used to adjust the sample sizes. The Supplemental materials (Section 3) and the Open Science Framework (https://osf.io/p68gm/) include the R code^36^ to calculate the minimum number of units to be added to the groups or therapists, and to individuals in the other arm to achieve the required power in a maximin design. In cluster randomized trials where outcome variances, intraclass correlations, cluster sizes, or number of clusters differ across arms, numerical evaluations show that for 80% or 90% power, with at least 8 clusters per arm, two additional clusters are needed for two-tailed tests at a 5% significance level and four for tests at a 1% level.^37^ In partially nested trials the intraclass correlation in one of the arms is zero, but since Candel et al.^37^ also considered intraclass correlations as small as 0.01, we expect this rule of thumb also to hold for partially nested trials, though in the arm without clustering the required increase applies to the number of subjects instead of the number of clusters.

Our results assumed equal group sizes or therapist caseloads. However, therapy groups often vary in participant numbers, and therapist caseloads can differ. Even if equal recruitment is achieved initially, dropout leads to varied group or therapist sample sizes during data analysis. Unequal group sizes or therapist caseloads in treatment *G* reduce efficiency and power. This can be repaired in an almost cost-efficient way, by recruiting more groups or therapists in one arm and increasing the number of individuals in the other by the same percentage. Let *CV* be the standard deviation of cluster sizes divided by the average cluster size in treatment *G*. If *CV* ≤ 0.6, adding 11% more clusters to one arm and 11% more individuals to the other has been shown to provide a sufficient correction.^38^ More generally, for cluster randomized trials, a formula has been developed to calculate the extra number of clusters needed to compensate for power loss from unequal cluster sizes.^28,39,40^ If $CV_{max}\;$ is the maximum expected *CV* for a treatment arm, then to compensate for efficiency loss, $CV_{max}^{2}/(4-CV_{max}^{2})\times 100\%$ clusters should be added to that arm. This correction is a safe approximation in most cases.^39,40^ For maximum likelihood estimation this formula has been shown to be more close to the actual efficiency loss than expressions based on cluster-size weighted and unweighted analysis of treatment effects.^39,40^ In this paper, clustering occurs only in one treatment arm, so only the part of $var({\hat{\beta }}_{1})$ in equation (3) for treatment *G* is affected by cluster size variation. This implies less efficiency loss for partially nested randomized trials than for cluster randomized trials, and the loss can be offset by adding $CV_{max}^{2}/(4-CV_{max}^{2})\times 100\%$ clusters to the arm with clusters only.

For the empirical illustration in Section 4 with $n_{G}\;\in \;\{4,5,6,7,8,9,10\}$ , assume $c/\;s_{G}=5$ and $\;s_{G}/s_{I}=10.$ For the maximin design obtained by the Shiny app (*K* = 25, $n_{G}$ = 4, $n_{I}$ = 299, see last paragraph of Section 7), adjusting for a *t*-test increases *K* to 27 and $n_{I}\;$ to 301 using the *R*-code in the Supplemental materials. Accounting for cluster size variation raises *K* further to 30 and $n_{I}$ to 332 (11% increase), while $n_{G}$ remains 4.

## 9 Conclusion and discussion

This paper presents optimal designs for trials with clustering in one arm and quantitative outcomes, minimizing research costs while achieving a desired power level. The designs assume data analysis with a linear mixed model with heterogeneous outcome variances and heterogeneous costs for the two arms. Since optimal designs require knowledge of parameters of the analysis model that are not known at the design stage, maximin designs are presented. Maximin designs guarantee a specified power level for plausible parameter ranges at the lowest cost and maximize, for a fixed research budget, power for the worst-case values of these parameters. Maximin designs are also developed with constraints on the number of clusters or cluster size. Sample size formulas are provided for all design types and implemented in an interactive R Shiny app. The formulas are based on a z-test assuming known variance components, but in practice variance components are unknown and a *t*-test will be done. For using a *t*-test instead of a *z*-test, a rule of thumb is provided to adjust the number of clusters and participants. Guidelines are also given to correct for power loss due to size variation between groups or caseloads.

In planning a study, it is useful to have some information on unknown model parameters. For IRGT trials there is an overview study documenting the intraclass correlations related to groups in psychotherapy trials^33^ and for trials involving individual psychotherapy there is an overview of intraclass correlations associated with therapists.^41^ However, to plan a maximin design, not only intraclass correlations are relevant, but also the ratio of outcome variances of one treatment versus the other. Researchers should thus be encouraged to report not only the intraclass correlation, but also the total outcome variance, for each treatment arm, thereby facilitating future planning of similar studies.

An assumption of IRGT trials and trials where therapists treat multiple persons is that persons are randomly assigned to groups or therapists after having been randomly allocated to one of two treatments. However, in the second stage of the assignment process nonrandom sorting of individuals into groups or therapists may occur. This may be because of self-assignment to groups or therapists or because geography or other practical constraints do not allow for randomly assigning an individual to a specific group or therapist.^42^ Such non-random allocation may be a source of additional outcome variance between groups or therapists on top of that caused by group dynamics or therapist effects. That in turn increases the standard error of the treatment effect estimate. Some strategies to mitigate these effects are discussed by Weiss et al.^42^

Our work presented maximin designs for trials with a quantitative outcome. For group treatment trials with binary outcomes, Moerbeek et al.^31^ derived optimal designs assuming fixed group sizes. Future research could extend this by developing maximin designs without fixed group sizes. Additionally, studies on three-level designs, where units are allocated to different treatments at the highest level,^43–45^ are relevant for settings where persons are assigned to (therapy) groups and these groups in turn are assigned to different therapists or counsellors. Some of these studies may involve partial nesting, with three-level nesting in one arm and no nesting in the other. Also, therapists, for example, might serve both groups in one arm and individuals in the arm with individual therapy.^46^ Further research into optimal and maximin designs for such nested trials, incorporating cost and outcome variance heterogeneity, would be valuable.

## Supplemental Material

sj-docx-1-smm-10.1177_09622802251409388 - Supplemental material for Efficient design of partially nested randomized trials: A maximin approach Supplemental material, sj-docx-1-smm-10.1177_09622802251409388 for Efficient design of partially nested randomized trials: A maximin approach by Math JJM Candel and Gerard JP van Breukelen in Statistical Methods in Medical Research

## Appendix A: Derivation of the optimal design for a two-treatment parallel randomized trial with clustering in one arm

The variance of the treatment effect estimate is given by (see eq. (8) of the main text):

(A1)
$$var({\hat{\beta }}_{1})=\left[((n_{G}-1)\rho +1)\;\frac{\psi }{n_{G}K}+\frac{1}{n_{I}}\right]\sigma_{\varepsilon I}^{2}.$$

Suppose the total budget $b=b_{G}+b_{I}$ is divided into budget $b_{G}$ assigned to treatment *G* and a budget $b_{I}$ assigned to treatment *I.* In the context of cluster randomized trials, where there is nesting in both arms, it can the shown that the optimal cluster size for treatment *G* is given by:^30^

(A2)
$$n_{G}^{opt}=\sqrt{\frac{\left(1-\rho \right)}{\rho }}\times \sqrt{\frac{c}{s_{G}}},$$

and the number of clusters for that treatment is given by $K=b_{G}/(c+n_{G}s_{G})$ . This also applies for a design with nesting in only one of the treatments. Given the budget function in eq. (4) of the main text, we have $n_{I}=\frac{b-K(c+n_{G}s_{G})}{s_{I}}=\frac{b-b_{G}}{s_{I}}$ , and replacing *K* and $n_{I}$ by their expressions in terms of $b_{G}$ yields the following expression of the variance of the treatment effect estimate:

(A3)
$$var({\hat{\beta }}_{1})=\left[((n_{G}-1)\rho +1)\;\frac{\psi }{n_{G}}\times \frac{\left(c+n_{G}s_{G}\right)}{b_{G}}+\frac{s_{I}}{\left(b-b_{G}\right)}\right]\sigma_{\varepsilon I}^{2}.$$

So the budget $b_{G}$ that minimizes the variance of the treatment effect, yields the optimal number of clusters for treatment *G* and the optimal number of persons for treatment *I*.

Taking the derivative of $var({\hat{\beta }}_{1})\;$ with respect to $b_{G}$ yields:

(A4)
$$\begin{array}{r} \left[((n_{G}-1)\rho +1)\;\frac{\psi }{n_{G}}\times \frac{-(c+n_{G}s_{G})}{b_{G}^{2}}+\frac{s_{I}}{{\left(b-b_{G}\right)}^{2}}\right]\sigma_{\varepsilon I}^{2},\text{ or}\\ \left[((n_{G}-1)\rho +1)\;\frac{\psi }{n_{G}}\times \frac{-(c+n_{G}s_{G}){\left(b-b_{G}\right)}^{2}}{b_{G}^{2}{\left(b-b_{G}\right)}^{2}}+\frac{s_{I}b_{G}^{2}}{b_{G}^{2}{\left(b-b_{G}\right)}^{2}}\right]\sigma_{\varepsilon I}^{2}. \end{array}$$

Let $u=((n_{G}-1)\rho +1)\;\frac{\psi }{n_{G}}(c+n_{I}s_{I})$ , then we can rewrite eq. (A4) as

(A5)
$$\left[\frac{-u{\left(b-b_{G}\right)}^{2}}{b_{G}^{2}{\left(b-b_{G}\right)}^{2}}+\frac{s_{I}b_{G}^{2}}{b_{G}^{2}{\left(b-b_{G}\right)}^{2}}\right]\sigma_{\varepsilon I}^{2}.$$

The derivative of eq. (A5) with respect to $b_{G}\;$ is 0, when $(s_{I}-u)b_{G}^{2}+2b_{G}bu-b^{2}u=0$ . Solving for $b_{G}$ yields, after some further rewriting, two solutions:

(A6)
$$\begin{array}{rl} b_{G} & =b\sqrt{u}\frac{\left(\sqrt{u}+\sqrt{s_{I}}\right)}{\left(u-s_{I}\right)}\;\text{and}\;b_{G}=b\sqrt{u}\frac{\left(\sqrt{u}-\sqrt{s_{I}}\right)}{\left(u-s_{I}\right)},\;\;\;\text{or}\\ b_{G} & =\frac{b\sqrt{u}}{\left(\sqrt{u}-\sqrt{s_{I}}\right)}\;\text{and}\;b_{G}=\frac{b\sqrt{u}}{\left(\sqrt{u}+\sqrt{s_{I}}\right)}\;. \end{array}$$

Taking the derivative of eq. (A5) with respect to $b_{G}$ , yields the second derivative of $var({\hat{\beta }}_{1})\;$ in eq. (A3) with respect to $b_{G}$ :

(A7)
$$\frac{2u}{b_{G}^{3}}+\frac{2s_{I}}{{\left(b-b_{G}\right)}^{3}}.$$

The second derivative is positive for the second solution in eq. (A6), $b_{G}=\frac{b\sqrt{u}}{\left(\sqrt{u}+\sqrt{s_{I}}\right)}$ , since both $b_{G}^{3}$ and $(b-b_{G}{)}^{3}$ are positive. So this solution for $b_{G}\;$ yields a minimum for the variance of the treatment effect estimate. The first solution $b_{G}=\frac{b\sqrt{u}}{\left(\sqrt{u}-\sqrt{s_{I}}\right)}$ in eq. (A6) can be shown to yield a negative second derivative, and thus yields a maximum for the variance of the treatment effect estimate.

Rewriting *u* in terms of the optimal cluster size in treatment *G* given in eq. (A2), we obtain:

(A8)
$$\begin{array}{rl} u & =\left(\rho +\frac{\left(1-\rho \right)}{n_{G}}\right)\;\psi (c+n_{G}s_{G})=\left(\rho +(1-\rho )\sqrt{\frac{\rho }{\left(1-\rho \right)}}\times \sqrt{\frac{s_{G}}{c}}\right)\;\psi \left(c+\sqrt{\frac{\left(1-\rho \right)}{\rho }}\times \sqrt{\frac{c}{s_{G}}}s_{G}\right)\\ & =\sqrt{\rho }\left(\sqrt{\rho }+\sqrt{\left(1-\rho \right)}\times \sqrt{\frac{s_{G}}{c}}\right)\;\psi \sqrt{\frac{c}{\rho }}\left(\sqrt{c\rho }+\sqrt{\left(1-\rho \right)}\times \sqrt{s_{G}}\right)\\ & =\left(\sqrt{c\rho }+\sqrt{\left(1-\rho \right)}\times \sqrt{s_{G}}\right)\;\psi \left(\sqrt{c\rho }+\sqrt{\left(1-\rho \right)}\times \sqrt{s_{G}}\right)\\ & ={\left(\sqrt{c\rho }+\sqrt{\left(1-\rho \right)}\times \sqrt{s_{G}}\right)}^{2}\;\psi . \end{array}$$

For the optimal $b_{G}=\frac{b\sqrt{u}}{\left(\sqrt{u}+\sqrt{s_{I}}\right)}$ *,* we thus obtain $b_{G}=\frac{b\left(\sqrt{c\rho }+\sqrt{\left(1-\rho \right)}\sqrt{s_{G}}\right)\;\sqrt{\psi }}{\left(\left(\sqrt{c\rho }+\sqrt{\left(1-\rho \right)}\sqrt{s_{G}}\right)\;\sqrt{\psi }+\sqrt{s_{I}}\right)}$ , and the optimal sample size for treatment *I* can be obtained as:

(A9)
$$\begin{array}{rl} n_{I}^{opt} & =\frac{b-b_{G}}{s_{I}}=\frac{b\left(\left(\sqrt{c\rho }+\sqrt{\left(1-\rho \right)}\sqrt{s_{G}}\right)\;\sqrt{\psi \;}+\text{\textbackslash{}; }\sqrt{s_{I}}-\left(\sqrt{c\rho }+\sqrt{\left(1-\rho \right)}\sqrt{s_{G}}\right)\;\sqrt{\psi }\right)}{\left(\left(\sqrt{c\rho }+\sqrt{\left(1-\rho \right)}\sqrt{s_{G}}\right)\;\sqrt{\psi }+\sqrt{s_{I}}\right)}\times \frac{1}{s_{I}}\\ & =\frac{b\sqrt{s_{I}}}{\left(\left(\sqrt{c\rho }+\sqrt{\left(1-\rho \right)}\sqrt{s_{G}}\right)\;\sqrt{\psi }+\sqrt{s_{I}}\right)}\times \frac{1}{s_{I}}=\frac{b}{\sqrt{s_{I}}\left(\left(\sqrt{c\rho }+\sqrt{\left(1-\rho \right)}\sqrt{s_{G}}\right)\;\sqrt{\psi }+\sqrt{s_{I}}\right)}, \end{array}$$

which is eq. (5) of the main text. Further, the optimal *K* can now be obtained as, also considering the optimal $n_{G}$ in eq. (A2):

(A10)
$$\begin{array}{rl} K^{opt} & =\frac{b_{G}}{c+n_{G}^{opt}s_{G}}=\frac{b\left(\sqrt{c\rho }+\sqrt{\left(1-\rho \right)}\sqrt{s_{G}}\right)\;\sqrt{\psi }}{\left(\left(\sqrt{c\rho }+\sqrt{\left(1-\rho \right)}\sqrt{s_{G}}\right)\;\sqrt{\psi }+\sqrt{s_{I}}\right)}\times \frac{1}{c+\sqrt{\frac{\left(1-\rho \right)}{\rho }}\times \sqrt{\frac{c}{s_{G}}}\times s_{G}}\\ & =\frac{b\left(\sqrt{c\rho }+\sqrt{\left(1-\rho \right)}\sqrt{s_{G}}\right)\;\sqrt{\psi }}{\left(\left(\sqrt{c\rho }+\sqrt{\left(1-\rho \right)}\sqrt{s_{G}}\right)\;\sqrt{\psi }+\sqrt{s_{I}}\right)}\times \frac{\sqrt{\rho }}{\sqrt{c}\left(\sqrt{c\rho }+\sqrt{\left(1-\rho \right)}\sqrt{s_{G}}\right)\;}\\ & =b\times \frac{\;\sqrt{\rho \psi }}{\sqrt{c}\left(\left(\sqrt{c\rho }+\sqrt{\left(1-\rho \right)}\sqrt{s_{G}}\right)\;\sqrt{\psi }+\sqrt{s_{I}}\right)}, \end{array}$$

as given by eq. (5) in the main text.

*Fixed cluster size* $n_{G}$ *: * In case the cluster size $n_{G}\;$ is fixed, only the number of clusters *K* and the number of subjects in treatment *I,* $n_{I}$ , can be optimized. The variance of the treatment effect estimate in eq. (3) is the same as the variance of the difference between the average of cluster means in treatment *G* and the average of individual outcomes in treatment *I*. The optimal allocation of clusters to *G* and of persons to *I,* then follows from the optimal allocation of a randomized controlled trial, where the units in treatment *G* are now clusters instead of individuals. Since the variance of a cluster mean is $\sigma_{0}^{2}+\sigma_{\varepsilon G}^{2}/n_{G}$ and the costs of a cluster are $c_{k}=c+n_{G}s_{G}$ , we obtain:^17^

(A11)
$$\begin{array}{r} \frac{K^{opt}}{n_{I}^{opt}}=\sqrt{\frac{\sigma_{0}^{2}+\sigma_{\varepsilon G}^{2}/n_{G}}{\sigma_{\varepsilon I}^{2}}}\times \sqrt{\frac{s_{I}}{c_{k}}}. \end{array}$$

Substituting the expression of $n_{I}^{opt}$ from eq. (A11) into the budget function in eq. (4) yields:

(A12)
$$\begin{array}{r} b=K^{opt}c_{k}+s_{I}K^{opt}\times \sqrt{\frac{\sigma_{\varepsilon I}^{2}}{\sigma_{0}^{2}+\sigma_{\varepsilon G}^{2}/n_{G}}}\times \sqrt{\frac{c_{k}}{s_{I}}}, \end{array}$$

which after solving for $K^{opt}$ yields:

(A13)
$$\begin{array}{r} K^{opt}=\frac{b}{\sqrt{c_{k}}}\left(\frac{\sqrt{\sigma_{0}^{2}+\sigma_{\varepsilon G}^{2}/n_{G}}}{\sqrt{c_{k}}\sqrt{\sigma_{0}^{2}+\sigma_{\varepsilon G}^{2}/n_{G}}+\sqrt{s_{I}}\sqrt{\sigma_{\varepsilon I}^{2}}}\right). \end{array}$$

Multiplying the numerator and denominator of eq. (A13) by $\sqrt{n_{G}}$ and dividing by $\sqrt{\sigma_{\varepsilon I}^{2}}$ , we obtain:

(A14)
$$\begin{array}{rl} K^{opt} & =\frac{b}{\sqrt{c_{k}}}\left(\frac{\sqrt{\frac{n_{G}\sigma_{0}^{2}+\sigma_{\varepsilon G}^{2}}{\sigma_{\varepsilon I}^{2}}}}{\sqrt{c_{k}}\sqrt{\frac{n_{G}\sigma_{0}^{2}+\sigma_{\varepsilon G}^{2}}{\sigma_{\varepsilon I}^{2}}}+\sqrt{s_{I}}\sqrt{n_{G}}}\right)=\frac{b}{\sqrt{c_{k}}}\left(\frac{\sqrt{\frac{(n_{G}-1)\sigma_{0}^{2}}{\sigma_{\varepsilon I}^{2}}+\frac{\sigma_{0}^{2}+\sigma_{\varepsilon G}^{2}}{\sigma_{\varepsilon I}^{2}}}\;}{\sqrt{c_{k}}\sqrt{\frac{(n_{G}-1)\sigma_{0}^{2}}{\sigma_{\varepsilon I}^{2}}+\frac{\sigma_{0}^{2}+\sigma_{\varepsilon G}^{2}}{\sigma_{\varepsilon I}^{2}}}+\sqrt{s_{I}}\sqrt{n_{G}}}\right),\;\;\text{or}\\ K^{opt} & =\frac{b}{\sqrt{c_{k}}}\left(\frac{\sqrt{\frac{\sigma_{0}^{2}+\sigma_{\varepsilon G}^{2}}{\sigma_{\varepsilon I}^{2}}}\sqrt{\frac{(n_{G}-1)\sigma_{0}^{2}}{\sigma_{0}^{2}+\sigma_{\varepsilon G}^{2}}+1}\;}{\sqrt{c_{k}}\sqrt{\frac{\sigma_{0}^{2}+\sigma_{\varepsilon G}^{2}}{\sigma_{\varepsilon I}^{2}}}\sqrt{\frac{(n_{G}-1)\sigma_{0}^{2}}{\sigma_{0}^{2}+\sigma_{\varepsilon G}^{2}}+1}+\sqrt{s_{I}}\sqrt{n_{G}}}\right). \end{array}$$

Since $\rho =\frac{\sigma_{0}^{2}}{\sigma_{0}^{2}+\sigma_{\varepsilon G}^{2}}$ , $\psi =(\sigma_{0}^{2}+\sigma_{\varepsilon G}^{2})/\sigma_{\varepsilon I}^{2}$ , and $c_{k}=c+n_{G}s_{G},\;$ we can rewrite eq. (A14) as

(A15)
$$\begin{array}{r} K^{opt}=\frac{b}{\sqrt{c+n_{G}s_{G}}}\left(\frac{\sqrt{\psi }\sqrt{(n_{G}-1)\rho +1}\;}{\sqrt{c+n_{G}s_{G}}\sqrt{\psi }\sqrt{(n_{G}-1)\rho +1}+\sqrt{s_{I}}\sqrt{n_{G}}}\right), \end{array}$$

which is the optimal number of clusters in eq. (8) of the main text.

Next substituting the optimal *K* in eq. (A13) into eq. (A11), yields:

(A16)
$$\begin{array}{rl} n_{I}^{opt} & =K^{opt}\times \sqrt{\frac{\sigma_{\varepsilon I}^{2}}{\sigma_{0}^{2}+\sigma_{\varepsilon G}^{2}/n_{G}}}\times \sqrt{\frac{c_{k}}{s_{I}}}=\frac{b}{\sqrt{c_{k}}}\left(\frac{\sqrt{\sigma_{0}^{2}+\sigma_{\varepsilon G}^{2}/n_{G}}}{\sqrt{c_{k}}\sqrt{\sigma_{0}^{2}+\sigma_{\varepsilon G}^{2}/n_{G}}+\sqrt{s_{I}}\sqrt{\sigma_{\varepsilon I}^{2}}}\right)\times \sqrt{\frac{\sigma_{\varepsilon I}^{2}}{\sigma_{0}^{2}+\sigma_{\varepsilon G}^{2}/n_{G}}}\times \sqrt{\frac{c_{k}}{s_{I}}}\\ & =\frac{b}{\sqrt{s_{I}}}\left(\frac{\sqrt{\sigma_{\varepsilon I}^{2}}}{\sqrt{c_{k}}\sqrt{\sigma_{0}^{2}+\sigma_{\varepsilon G}^{2}/n_{G}}+\sqrt{s_{I}}\sqrt{\sigma_{\varepsilon I}^{2}}}\right) \end{array}$$

Multiplying the numerator and denominator of eq. (A16) by $\sqrt{n_{G}}$ and dividing by $\sqrt{\sigma_{\varepsilon I}^{2}}$ respectively, yields:

(A17)
$$\begin{array}{r} n_{I}^{opt}=\frac{b}{\sqrt{s_{I}}}\left(\frac{\sqrt{\sigma_{\varepsilon I}^{2}n_{G}}}{\sqrt{c_{k}}\sqrt{n_{G}\sigma_{0}^{2}+\sigma_{\varepsilon G}^{2}}+\sqrt{s_{I}}\sqrt{\sigma_{\varepsilon I}^{2}n_{G}}}\right)=\frac{b}{\sqrt{s_{I}}}\left(\frac{\sqrt{n_{G}}}{\sqrt{c_{k}}\sqrt{\frac{n_{G}\sigma_{0}^{2}+\sigma_{\varepsilon G}^{2}}{\sigma_{\varepsilon I}^{2}}}+\sqrt{s_{I}}\sqrt{n_{G}}}\right), \end{array}$$

which can be further elaborated as

(A18)
$$\begin{array}{r} n_{I}^{opt}=\frac{b}{\sqrt{s_{I}}}\left(\frac{\sqrt{n_{G}}}{\sqrt{c_{k}}\sqrt{\frac{\sigma_{0}^{2}+\sigma_{\varepsilon G}^{2}}{\sigma_{\varepsilon I}^{2}}}\sqrt{\frac{(n_{G}-1)\sigma_{0}^{2}}{\sigma_{0}^{2}+\sigma_{\varepsilon G}^{2}}+1}+\sqrt{s_{I}}\sqrt{n_{G}}}\right)=\frac{b}{\sqrt{s_{I}}}\left(\frac{\sqrt{n_{G}}}{\sqrt{c+n_{G}s_{G}}\sqrt{\psi }\sqrt{\rho (n_{G}-1)+1}+\sqrt{s_{I}}\sqrt{n_{G}}}\right), \end{array}$$

which is the optimal sample size for treatment *I* in eq. (8) of the main text.

*Fixed number of clusters K:* The variance of the treatment effect estimate is:

(A19)
$$\begin{array}{r} var({\hat{\beta }}_{1})=\frac{\sigma_{0}^{2}}{K\;}+\frac{\sigma_{\varepsilon G}^{2}}{Kn_{G}}+\frac{\sigma_{\varepsilon I}^{2}}{n_{I}}. \end{array}$$

Since the number of clusters *K* is fixed, and thus $\frac{\sigma_{0}^{2}}{K\;}$ is fixed, minimizing the variance in eq. (A19) reduces to minimizing the rightmost part:

(A20)
$$\begin{array}{r} \frac{\sigma_{\varepsilon G}^{2}}{Kn_{G}}+\frac{\sigma_{\varepsilon I}^{2}}{n_{I}}. \end{array}$$

Eq. (A20) is the expression for the variance of the treatment effect estimate in a randomized controlled trial, where the number of subjects in one arm is $Kn_{G}$ and $n_{I}$ in the other, and the relevant budget function is $b-Kc=n_{G}s_{G}\;$ + $n_{I}s_{I}$ . For such a design the optimal allocation ratio can be shown to be:^17^

(A21)
$$\begin{array}{r} \frac{n_{G}^{opt}K}{n_{I}^{opt}}=\sqrt{\frac{\sigma_{\varepsilon G}^{2}}{\sigma_{\varepsilon I}^{2}}}\times \sqrt{\frac{s_{I}}{s_{G}}}. \end{array}$$

The budget function for the optimal design can now be written as

(A22)
$$\begin{array}{r} b-Kc\;=n_{G}^{opt}Ks_{G}+n_{G}^{opt}K\sqrt{\frac{\sigma_{\varepsilon I}^{2}}{\sigma_{\varepsilon G}^{2}}}\times \sqrt{\frac{s_{G}}{s_{I}}}s_{I}=n_{G}^{opt}K\sqrt{s_{G}}\left(\frac{\sqrt{s_{G}}\sigma_{\varepsilon G}+\sqrt{s_{I}}\sigma_{\varepsilon I}}{\sigma_{\varepsilon G}}\right), \end{array}$$

which yields the following optimal solution for $n_{G}$

(A23)
$$\begin{array}{r} n_{G}^{opt}=\frac{(b-Kc)\sigma_{\varepsilon G}}{K\sqrt{s_{G}}\left(\sqrt{s_{G}}\sigma_{\varepsilon G}+\sqrt{s_{I}}\sigma_{\varepsilon I}\right)}. \end{array}$$

Substituting $n_{G}^{opt}$ into eq. (A21) and solving for $n_{I},\;$ then gives the optimal $n_{I}$ :

(A24)
$$\begin{array}{r} n_{I}^{opt}=\frac{(b-Kc)\sigma_{\varepsilon I}}{\sqrt{s_{I}}\left(\sqrt{s_{G}}\sigma_{\varepsilon G}+\sqrt{s_{I}}\sigma_{\varepsilon I}\right)}. \end{array}$$

Noting that $\rho =\frac{\sigma_{0}^{2}}{\sigma_{0}^{2}+\sigma_{\varepsilon G}^{2}}$ and $\psi =(\sigma_{0}^{2}+\sigma_{\varepsilon G}^{2})/\sigma_{\varepsilon I}^{2}$ , these optimal cluster sizes can be rewritten as eq. (10) of the main text. 
