# Supplementary material for: Efficient design of partially nested randomized trials: A maximin approach
Source: Stat Methods Med Res. 2026 Mar 13;35(4):695–712. doi: 10.1177/09622802251409388 (PMC13161498; doi:10.1177/09622802251409388)
Supplement: sj-docx-1-smm-10.1177_09622802251409388 - Supplemental material for Efficient design of partially nested randomized trials: A maximin approach [file sj-docx-1-smm-10.1177_09622802251409388.docx]

**Supplementary file**

[Section 1: Optimal allocation ratio for a randomized trial with clustering in one arm 1](#_Toc183365041)

[Section 2: Effect of the variance ratio on required budget of the maximin design with fixed cluster sizes relative to the required budget of the unrestricted maximin design 2](#_Toc183365042)

[Section 3: R-code to correct the number of clusters in one arm and number of individuals in the other arm for a maximin trial with clustering in one arm 4](#_Toc183365043)

# Section 1: Optimal allocation ratio for a randomized trial with clustering in one arm

From eq. (5) of the main text it follows that the optimal allocation ratio for the number of clusters in treatment arm *G* versus the number of individuals in treatment *I* is equal to:

$\frac{K^{opt}}{n_{I}^{opt}}=\sqrt{\frac{s_{I}}{c}}\times\sqrt{\rho\psi}=\sqrt{\frac{s_{I}}{c}}\times\sqrt{\frac{\sigma_{0}^{2}}{\sigma_{\varepsilon I}^{2}}}$. (1.1)

We will show that the optimal allocation ratio in eq. (1.1) equals the square root of the variance of the cluster averages in *G* divided by the variance of individual scores, multiplied by the square root of the costs of an individual in *I* divided by the costs of a cluster in *G*. In formula:

$\frac{K^{opt}}{n_{I}^{opt}}=\sqrt{\frac{var\left( {\sum_{i=1}^{n_{G}^{opt}} y_{ij}}/{n_{G}^{opt}} \right)}{\sigma_{\varepsilon I}^{2}}}\times\sqrt{\frac{s_{I}}{c+n_{G}^{opt}s_{G}}=}\sqrt{\frac{\sigma_{0}^{2}+\frac{\sigma_{\varepsilon G}^{2}}{n_{G}^{opt}}}{\sigma_{\varepsilon I}^{2}}}\times\sqrt{\frac{s_{I}}{c+n_{G}^{opt}s_{G}}}$ . (1.2)

The optimal cluster size for *G* is given by (see eq. (5) of main text) $n_{G}^{opt}=\sqrt{\frac{c}{s_{G}}}\times\frac{\sigma_{\varepsilon G}}{\sigma_{0}}$ , implying that $\frac{\sigma_{\varepsilon G}^{2}}{n_{G}^{opt}}=\sigma_{\varepsilon G}\sigma_{0}\sqrt{\frac{s_{G}}{c}}$. The leftmost part of the multiplication in eq. (1.2) can be now rewritten as follows:

$\sqrt{\frac{\sigma_{0}^{2}+\frac{\sigma_{\varepsilon G}^{2}}{n_{G}^{opt}}}{\sigma_{\varepsilon I}^{2}}}=\sqrt{\frac{\sigma_{0}^{2}+\sigma_{\varepsilon G}\sigma_{0}\sqrt{\frac{s_{G}}{c}}}{\sigma_{\varepsilon I}^{2}}}=\sqrt{\frac{{\sqrt{c}\sigma}_{0}^{2}+\sigma_{\varepsilon G}\sigma_{0}\sqrt{s_{G}}}{\sqrt{c}\sigma_{\varepsilon I}^{2}}}=\sqrt{\sigma_{0}}\sqrt{\frac{{\sqrt{c} \sigma}_{0}+\sigma_{\varepsilon G}\sqrt{s_{G}}}{\sqrt{c}\sigma_{\varepsilon I}^{2}}}$ . (1.3)

The optimal cluster size for *G* (eq. (5) of the main text) also implies that $n_{G}^{opt}s_{G}=\sqrt{cs_{G}}\times\frac{\sigma_{\varepsilon G}}{\sigma_{0}}$, and the rightmost part of the multiplication in eq. (1.2) can be elaborated as:

$\sqrt{\frac{s_{I}}{c+n_{G}^{opt}s_{G}}}=$ $\sqrt{\frac{s_{I}}{c+\sqrt{cs_{G}}\times\frac{\sigma_{\varepsilon G}}{\sigma_{0}}}}=\sqrt{\frac{s_{I}\sigma_{0}}{c\sigma_{0}+\sqrt{cs_{G}}\times\sigma_{\varepsilon G}}}=\sqrt{\frac{s_{I}\sigma_{0}}{\sqrt{c}\left( {\sqrt{c}\sigma}_{0}+\sigma_{\varepsilon G}\sqrt{s_{G}} \right)}}$. (1.4)

Based on eq. (1.3) and eq. (1.4), we can rewrite the optimal allocation ratio in eq. (1.2) as:

$\frac{K^{opt}}{n_{I}^{opt}}=\sqrt{\sigma_{0}}\sqrt{\frac{{\sqrt{c} \sigma}_{0}+\sigma_{\varepsilon G}\sqrt{s_{G}}}{\sqrt{c}\sigma_{\varepsilon I}^{2}}}\times\sqrt{\frac{s_{I}\sigma_{0}}{\sqrt{c}\left( {\sqrt{c}\sigma}_{0}+\sigma_{\varepsilon G}\sqrt{s_{G}} \right)}}=\sqrt{\frac{s_{I}}{c}}\times\sqrt{\frac{\sigma_{0}^{2}}{\sigma_{\varepsilon I}^{2}}}$, (1.5)

which is the same as eq. (1.1). From eq. (1.5), also follows eq. (6) in the main text:

$\frac{n_{G}^{opt}K^{opt}}{n_{I}^{opt}}=\sqrt{\frac{c}{s_{G}}}\times\frac{\sigma_{\varepsilon G}}{\sigma_{0}}\times\sqrt{\frac{s_{I}}{c}}\times\sqrt{\frac{\sigma_{0}^{2}}{\sigma_{\varepsilon I}^{2}}}=\sqrt{\frac{s_{I}}{s_{G}}} \times\frac{\sigma_{\varepsilon G}}{\sigma_{\varepsilon I}}$ . (1.6)

# Section 2: Effect of the variance ratio on required budget of the maximin design with fixed cluster sizes relative to the required budget of the unrestricted maximin design

If ${c^{o}=c+n}_{G}s_{G}$, then the ratio of the budget of the maximin design with $n_{G}$ fixed (see eq. (9)) relative to the budget of the maximin design without restrictions on the cluster sizes (see eq. (7)) can be expressed as:

$\frac{b(n_{G} fixed)}{b(unrestricted)}=\frac{\left( \sqrt{\psi_{max}c^{o}} \sqrt{\rho_{max}+\frac{\left( 1-\rho_{max} \right)}{n_{G}}} + \sqrt{s_{I}} \right)^{2}}{\left( \sqrt{\psi_{max}\rho_{max}c}+\sqrt{\psi_{max}s_{G}\left( 1-\rho_{max} \right)} + \sqrt{s_{I}} \right)^{2}}=\frac{\left( f(\psi_{max}) \right)^{2}}{\left( g(\psi_{max}) \right)^{2}}$ . (2.1)

We want to show that $\frac{\partial}{\partial\psi_{max}}\left( \frac{\left( f(\psi_{max}) \right)^{2}}{\left( g(\psi_{max}) \right)^{2}} \right)\geq0$. Since $f\left( \psi_{max} \right)>0$ and $g\left( \psi_{max} \right)>0$, one can derive that the ratio in (2.1) increases in $\psi_{max}$ if $f^{'}\left( \psi_{max} \right)g\left( \psi_{max} \right) \geq f\left( \psi_{max} \right)g^{'}\left( \psi_{max} \right)$. So, if

$\frac{1}{2}\frac{1}{\sqrt{\psi_{max}}}\sqrt{c^{o}}\sqrt{\rho_{max}+\frac{\left( 1-\rho_{max} \right)}{n_{G}}}\left( \sqrt{\psi_{max}\rho_{max}c}+\sqrt{\psi_{max}s_{G}\left( 1-\rho_{max} \right)} + \sqrt{s_{I}} \right)\geq$

$\left( \sqrt{\psi_{max}c^{o}}\sqrt{\rho_{max}+\frac{\left( 1-\rho_{max} \right)}{n_{G}}}+ \sqrt{s_{I}} \right)\frac{1}{2}\frac{1}{\sqrt{\psi_{max}}}\left( \sqrt{c\rho_{max}}+\sqrt{s_{G}\left( 1-\rho_{max} \right)} \right)$ ,

Or

$\sqrt{{\psi_{max}c}^{o}}\sqrt{\rho_{max}+\frac{\left( 1-\rho_{max} \right)}{n_{G}}}\left( \sqrt{c\rho_{max}}+\sqrt{s_{G}\left( 1-\rho_{max} \right)} \right)+ \sqrt{s_{I}}\sqrt{c^{o}}\sqrt{\rho_{max}+\frac{\left( 1-\rho_{max} \right)}{n_{G}}}\geq$

$\sqrt{\psi_{max}c^{o}}\sqrt{\rho_{max}+\frac{\left( 1-\rho_{max} \right)}{n_{G}}}\left( \sqrt{c\rho_{max}}+\sqrt{s_{G}\left( 1-\rho_{max} \right)} \right)+ \sqrt{s_{B}}\left( \sqrt{c\rho_{max}}+\sqrt{s_{G}\left( 1-\rho_{max} \right)} \right)$ ,

or

$\sqrt{s_{I}}\sqrt{c^{o}}\sqrt{\rho_{max}+\frac{\left( 1-\rho_{max} \right)}{n_{G}}}\geq\sqrt{s_{I}}\left( \sqrt{c\rho_{max}}+\sqrt{s_{G}\left( 1-\rho_{max} \right)} \right)$ ,

or

$\left( {c+n}_{G}s_{G} \right)\left( \rho_{max}+\frac{\left( 1-\rho_{max} \right)}{n_{G}} \right)\geq c\rho_{max}+s_{G}\left( 1-\rho_{max} \right)+2\sqrt{c\rho_{max}}\sqrt{s_{G}\left( 1-\rho_{max} \right)}$ ,

or

$c\rho_{max}{+ n}_{G}s_{G}\rho_{max}+c\left( \frac{\left( 1-\rho_{max} \right)}{n_{G}} \right)+s_{G}\left( 1-\rho_{max} \right)\geq c\rho_{max}+s_{G}\left( 1-\rho_{max} \right)+2\sqrt{c\rho_{max}}\sqrt{s_{G}\left( 1-\rho_{max} \right)}$

or

$n_{G}s_{G}\rho_{max}+c\left( \frac{\left( 1-\rho_{max} \right)}{n_{G}} \right)\geq2\sqrt{c\rho_{max}}\sqrt{s_{G}\left( 1-\rho_{max} \right)}$ . (2.2)

After multiplication of both sides of the inequality in eq (2.2) by $n_{G}$

$n_{G}^{2}s_{G}\rho_{max}+c\left( 1-\rho_{max} \right)-2n_{G}\sqrt{c\rho_{max}}\sqrt{s_{G}\left( 1-\rho_{max} \right)}\geq0$ . (2.3)

After division of eq. (2.3) by $s_{G}$and $\rho_{max}$

$n_{G}^{2}+\frac{c}{s_{G}}\frac{\left( 1-\rho_{max} \right)}{\rho_{max}}-2n_{G}\sqrt{\frac{c}{s_{G}}}\sqrt{\frac{\left( 1-\rho_{max} \right)}{\rho_{max}}}\geq0$,

which is

$n_{G}^{2}+\left( n_{G}^{mmd} \right)^{2}-2n_{G}n_{G}^{mmd}\geq0$, or $\left( n_{G}-n_{G}^{mmd} \right)^{2}\geq0$, which is true.

# Section 3: R-code to correct the number of clusters in one arm and number of individuals in the other arm for a maximin trial with clustering in one arm

For randomized trials with clustering in one arm, where the cluster sizes are the same and no covariates are involved, the data can also be analysed by an independent samples *t*-test on the cluster means for one arm and the individual outcomes for the other arm. Let *t(df, 1-*$\alpha/2$*)* denote the 100(1-$\alpha/2)$th percentile of the central *t*-distribution with *df* degrees of freedom. For a two-sided test on whether the two treatments have the same mean or not, the null hypothesis of no effect is rejected if the *t*-statistic is above *t(df, 1-*$\alpha/2$*)* or below *t(df,* $\alpha/2)$*.* The degrees of freedom are given by the Satterthwaite approximation, translated to a scenario where the scores are cluster averages in one arm and the individual scores in the other, and the sample sizes for the treatments are the numbers of clusters in one arm and the number of individuals in the other arm. If there is a treatment effect the *t*-statistic is *t*-distributed with *df* degrees of freedom and non-centrality parameter $\lambda$, *T(df,* $\lambda$*).* The non-centrality parameter $\lambda$ is ${\beta_{1}}/{\sqrt{Var( \hat{\beta_{1}})}}$, where $\beta_{1}$ is the true treatment effect and $Var( \hat{\beta_{1}})$ is given by eq. (3) of the main text. For a two-sided test at a type I error rate $\alpha$ on whether the two treatment means are equal or not, the general formula for the power is now given by:

$P\left[ T\left( df,\lambda\right)< t(df, \alpha/2) \right]+ P\left[ T\left( df,\lambda\right)> t(df, 1-\alpha/2) \right]$*.* (3.1)

Below an R-code is provided that finds the smallest number of clusters and individuals to be added to treatment *G* and treatment *I* respectively in the maximin design such that the power according to the *t*-distribution in eq. (3.1) is above the required power level.

**### R-code to correct for using the two-sided z-test in sample size calculation**

**### Parallel randomized trial with clustering in one arm (G) and no clustering in the**

**### other (I)**

### The correction in sample size is, by grid search, examined for

### all rhoG and psi within their plausible ranges, as the

### correction may be so large for some of the parameter values,

### that the required number of clusters for G and individuals for I

### (K and nI), exceed the corrected K and nI needed for the

### maximin values of these parameters.

### Output is the corrected number of clusters of one arm (K),

### the original number of individuals per cluster in G (nG),

### and the corrected number of individuals in the other arm (nI)

#################################

## START of required user input #

#################################

rm(list=ls(all=TRUE))

## ES = (average outcome in G - average outcome in I)/sqrt(outcome

## variance in I)

ES = 0.5

## psi = outcome variance(G)/outcome variance(I)

## specify minimum and maximum of plausible range

psimax = 1.5

psimin = 0.5

## rhoG = intraclass correlation in treatment G

## specify minimum and maximum of plausible range of rhoG

rhoGmin = 0.01

rhoGmax = 0.30

# type I error rate (alpha) and required power level (power_crit)

alpha = 0.05

power_crit= 0.80

# sample sizes calculated for the maximin design

Kstart = 25

nG = 4

nIstart = 299

#################################

## END of required user input #

#################################

### initialize the matrix with corrections for a grid of

### parameters combinations (501 x 501)

corr_m <- matrix( rep( c(-1), times=501*501), nrow =501, ncol =501 )

### initialize the matrix with power levels for a grid of

### parameters combinations (501 x 501)

power_m <- matrix( rep( c(0.0), times=501*501), nrow =501, ncol=501)

for (i1 in 0:500) {

psi = psimin + (psimax-psimin)*i1/500

for (i2 in 0:500) {

rhoG = rhoGmin + (rhoGmax-rhoGmin)*i2/500

# For the noncentrality parameter the outcome variances for

# treatment G and I are irrelevant given ES, and depends only on the

# ratio of these variances, psi.

# The degrees of freedom of the t-test also only depend on the

# ratio the variances for treatment G and I, psi.

# For convenience we set the outcome variance in I to 1.

# So: variance of the individual scores for I =1

s2kw = 1

# variance of the cluster means for G

s1kw = (rhoG + (1-rhoG)/nG)*psi*s2kw

K = Kstart -1

nI = nIstart -1

while (power_m[i1+1,i2+1] < power_crit) {

# start with correction term 0

corr_m[i1+1,i2+1] <- corr_m[i1+1,i2+1] + 1

# start with K and nI from the maximin design

K <- K + 1

nI <- nI + 1

# degrees of freedom (df) for the independent samples t-test;

# because of unequal variances the Satterthwaite approximation is

# used

dft <- (s1kw/K + s2kw/nI)^2

dfn <- s1kw^2/(K^2*(K-1)) + s2kw^2/(nI^2*(nI-1))

df <- dft/dfn

# critical value for t-distribution under the null hypothesis of

# no effect for a two-sided t-test

T_crit = qt(1-alpha/2, df = df, lower.tail = TRUE, log.p = FALSE)

# noncentrality parameter of t-distribution when there is an

# effect

ncp = ES/sqrt( (rhoG*(nG-1) +1)*psi/nG*(1/K) +1/nI)

# calculation of power for a corrected sample size

power_m[i1+1,i2+1] <- pt( - T_crit, df= df, ncp=ncp, lower.tail = TRUE, log.p = FALSE) + 1- pt( T_crit, df= df, ncp=ncp, lower.tail = TRUE, log.p = FALSE)

}

}}

### determine the maximum correction term and corrected number of

### clusters for G and corrected number of individuals for I

correction <- max(corr_m)

K = Kstart + correction

nI = nIstart + correction

cat("K = ", K, "; nG = ", nG, "; nI = ", nI, "\n")
